# Depression in sleep disturbance: A review on a bidirectional relationship, mechanisms and treatment

**DOI:** 10.1111/jcmm.14170

**Published:** 2019-02-07

**Authors:** Hong Fang, Sheng Tu, Jifang Sheng, Anwen Shao

**Affiliations:** ^1^ State Key Laboratory for Diagnosis and Treatment of Infectious Diseases, Collaborative Innovation Center for Diagnosis and Treatment of Infectious Diseases, The First Affiliated Hospital, College of Medicine Zhejiang University Hangzhou City, Zhejiang Province China; ^2^ Department of Neurosurgery Second Affiliated Hospital School of Medicine, Zhejiang University Hangzhou City, Zhejiang Province China

**Keywords:** bidirectional relation, depression, mechanism, sleep disturbance, treatment

## Abstract

Sleep disturbance is the most prominent symptom in depressive patients and was formerly regarded as a main secondary manifestation of depression. However, many longitudinal studies have identified insomnia as an independent risk factor for the development of emerging or recurrent depression among young, middle‐aged and older adults. This bidirectional association between sleep disturbance and depression has created a new perspective that sleep problems are no longer an epiphenomenon of depression but a predictive prodromal symptom. In this review, we highlight the treatment of sleep disturbance before, during and after depression, which probably plays an important role in improving outcomes and preventing the recurrence of depression. In clinical practice, pharmacological therapies, including hypnotics and antidepressants, and non‐pharmacological therapies are typically applied. A better understanding of the pathophysiological mechanisms between sleep disturbance and depression can help psychiatrists better manage this comorbidity.

## 1 INTRODUCTION

Currently, the problem of sleep disturbance has plagued nearly a quarter of the world's population. People who suffer from sleep problems throughout the year are more likely to have mental disorders such as bipolar disorder, generalized anxiety disorder, suicidal ideation and especially depression. Depressive disorders are one of the most commonly diagnosed psychiatric disorders, with a lifetime prevalence of approximately 16%.^1^, ^2^ Changes in sleep neurophysiology are often observed in depressive patients, and impaired sleep is, in many cases, the chief complaint of depression.^3^, ^4^ In the past, sleep disturbances were always regarded as a concomitant of depression, and sleep problems were seldom a treatment target given the general assumption that sleep disturbances would resolve as an associated symptom with the treatment of depression. Recently, there has been a great deal of evidence suggesting that sleep disturbances precede depression.^5^, ^6^ Depressed patients with sleep disturbance are likely to present more severe symptoms and difficulties in treatment.^7^ In addition, persistent insomnia is the most common residual symptom in depressed patients and is considered a vital predictor of depression relapse and may contribute to unpleasant clinical outcomes.^7^, ^8^ We now consider sleep disturbance as an independent diagnostic entity that may precipitate the onset of depressive disorder. Improving sleep is to improve outcomes of depression.^9^, ^10^ However, in clinical practice, only approximately half of depressive patients will seek treatment and nearly three quarters of people with depression will relapse at some point in their life.^11^, ^12^ These findings underscore the stringent need to prioritize prevention, rather than treatment, which means the proper handling of sleep disturbance before depression occurs. Antidepressant drugs and hypnotics are widely used for the treatment of patients with coincident depression and sleep complaints. However, some kinds of antidepressants may cause or even worsen sleep disturbances, and hypnotics always require consideration of drug dependence and resistance. Some non‐pharmacological treatments (eg cognitive behavioural therapy [CBT] and deep brain stimulation [DBS]) will be discussed below and have proven useful in such patients. Moreover, a good understanding of potential mechanisms between depression and sleep disturbance will be quite helpful in the treatment and prevention of these conditions. Here, we review and focus on the bidirectional connections, potential interactive mechanisms and therapeutic strategy for depression in sleep disturbance.

## 2 BIDIRECTIONAL RELATIONSHIP BETWEEN SLEEP DISORDERS AND DEPRESSION

Sleep disorders are a major health issue consisting of difficulties in various patterns and aspects of sleep that are often comorbid with mental disorders, for example, major depression disorder (MDD), bipolar disorder, post‐traumatic stress disorder and generalized anxiety disorder.^13^ Depression is one of the most prevalent mental health conditions and is estimated to be the leading cause of disease burden in the world by 2030.^14^, ^15^, ^16^ In depressive patients, sleep complaints (eg insomnia, narcolepsy, sleep disordered breathing and restless legs syndrome [RLS]) are universal in approximately 90% of patients.^17^ It is well known that sleep disturbances have been considered the core secondary symptom of depression in the past decades. Depression was usually regarded as a risk factor for developing insomnia.^18^ However, many longitudinal studies have demonstrated that insomnia is not only a prodromal manifestation of depression but also an independent risk factor for subsequent depression. The Johns Hopkins Precursors Study focused on the relationship between sleep disturbance and subsequent depression.^19^ In this study, insomnia in young men was considered a significant risk factor for subsequent depression and persisted for at least 30 years. The same conclusion was observed in other studies in which insomnia was highly related to subsequent depression among both young adults and old adults.^5^, ^20^ Patients with depression have abnormalities in sleep parameters across all phases of sleep architecture. The alterations in REM sleep are the most evident sleep characteristic in patients with depression, and those changes are typically regarded as a distinctive biological marker of depression.^21^ Thus, the relationship between depression and insomnia is conflicting according to all these studies. Theoretically, this would indicate that the association between depression and insomnia is not simply a cause–effect relationship, but instead a complex bidirectional one.

## 3 POTENTIAL MECHANISMS BETWEEN SLEEP DISTURBANCES AND DEPRESSION

### 3.1 Inflammation hypothesis

**Figure 1:**
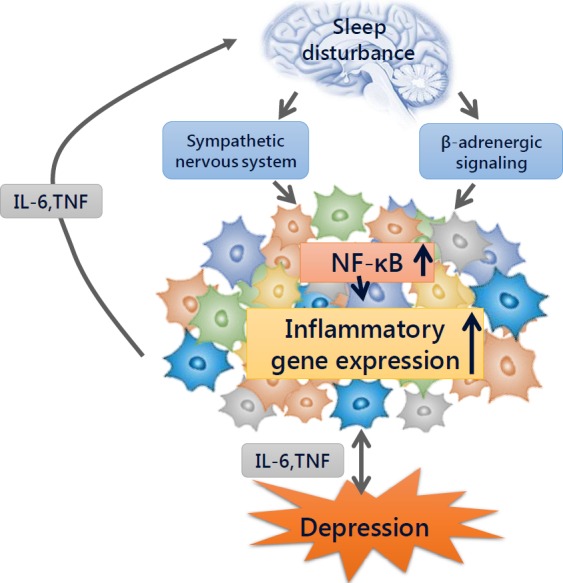
The inflammation mechanism between sleep disturbance and depression. Sleep disturbance may activate the sympathetic nervous system and β‐adrenergic signalling, which can release neuromediators and activate nuclear factor (NF)‐κB mediated inflammatory programs. Then NF‐κB will increase inflammatory cytokines, such as IL‐6 and TNF, by activating the expression of inflammatory genes. These inflammatory cytokines are highly correlated with the occurring of depression disorders, meanwhile, inflammatory activity in turn can influence sleep, but the specific interacting mechanisms remain unknown

It has been found that sleep deficiency contributes to increased levels of inflammatory cytokines (eg IL‐6 and TNF) throughout the day.^22^ In addition, evidence has shown that elevated levels of CRP and IL‐6 were associated with sleep impairment.^23^, ^24^, ^25^, ^26^, ^27^, ^28^ In general, sleep loss may cause the elevation of cellular inflammation, and these effects are more obvious in women.^29^, ^30^ By activating nuclear factor‐kappaB (NF‐κB), a key transcriptional control pathway in the inflammatory signalling cascade, sleep loss increases the transcription of IL‐6 and TNF.^31^ Meanwhile, a strong relationship between inflammation and depression has also been observed.^32^ In depressed patients, markers of inflammation have been shown to be higher than in non‐depressed individuals, and in patients with an inflammatory disorder, comorbid depression has been shown to be high.^33^ In addition, antagonism of endogenous inflammation has been effective in reducing depressive symptoms.^34^ In sum, sleep loss may increase markers of inflammation (eg IL‐6 and CRP) by activating the sympathetic nervous system and β‐adrenergic signalling, which can increase NF‐κB and activate inflammatory gene expression.^35^ Although the strong relation between sleep disturbance, inflammation and depression is obvious, the precise interaction between them remains unclear (Figure 1).

### 3.2 Biochemical pathways

Major depressive disorder has been associated with the disruption of REMS.^36^, ^37^ The transition into REM sleep is accompanied by a rapid decrease in monoamines (eg serotonin [5‐HT], norepinephrine [NE] and dopamine) and a concomitant increase in cholinergic tone.^38^ Thus, the mutual effect of cholinergic and monoaminergic neurons regulates the onset of REM sleep.^39^ Regarding the pathophysiology of depression, the monoamine hypothesis is the most known hypothesis, which assumes that alterations in the levels of monoamines are the cause of depression. In patients diagnosed with MDD, levels of serotonin metabolites and NE have been shown to be decreased, and abnormal genetic regulation of serotonergic transmission has been observed.^40^, ^41^ These monoamine neurotransmitters do not operate in isolation but are integrally interconnected.^42^ In fact, some kinds of antidepressant drugs, such as tricyclic antidepressants (TCA), selective serotonin reuptake inhibitors (SSRI), norepinephrine reuptake inhibitors (NRI) and serotonin‐norepinephrine reuptake inhibitors (SNRI), can improve depressive symptoms in a way that validates this hypothesis.^43^ Notably, the dysregulation of these same monoamine neurotransmitters that are responsible for the REM sleep abnormalities, is also related to the presentation of depression. However, some studies have found that there is no causal relation between REM sleep abnormalities and depression, which indicates that there may be two or more parallel pathways for the regulation of REM sleep and depression.^44^ The role of monoamines and other related neurotransmitters should be better elucidated for understanding the association between sleep and depression.

### 3.3 Genetic correlations

It was thought that insomnia was induced by environmental factors, stress or other mental diseases, but recent evidence has shown that genetic factors may also be involved. Twin studies have indicated that insomnia is heritable in adults.^45^ A recent large longitudinal study confirmed genetic influences on insomnia symptoms.^46^ In addition, twin studies demonstrated that MDD is also heritable, with moderate to high levels of heritability.^47^, ^48^ Moreover, insomnia and depression are not only clinically related but also genetically related. Complete genetic overlap between insomnia and MDD was reported in several studies.^49^, ^50^ Another study focused on children found that the genetic correlation between sleep and depression was 0.64.^51^ However, some studies have demonstrated only low or non‐significant correlations between depression and sleep.^52^, ^53^, ^54^ A subsequent well‐designed study concluded that the latent additional genetic influences on insomnia overlapped significantly (56% for females, 74% for males) with MDD.^55^ In other words, those papers have suggested that the genes influencing insomnia also influence depression. However, at the moment, we know only that insomnia and depression are partly related by genetics. Further studies focused on particular genes that are involved in the specific gene regulations in insomnia and depression and exactly how these genes function is required.

### 3.4 Circadian rhythm

Circadian rhythm is a 24‐hour rhythm in physiology and behaviour controlled by molecular clocks in suprachiasmatic nuclei (SCN). The circadian system plays an important role in sleep/wake cycle regulation, including sleep duration, continuity and architecture.^56^ The mechanisms underlying circadian regulation are cell self‐sustained transcription‐translation feedback loops regulating expression of a wide array of clock genes.^57^, ^58^ These intrinsically rhythmic cells are mostly concentrated in SCN. The pineal gland is an important downstream projection of the SCN by secreting melatonin, which is a crucial factor in the initiation of sleep. By interacting with circadian rhythm, the duration and architecture of sleep is at meanwhile regulated by process homeostatic which is based on the history of sleep/wake cycles.^59^ A marked disruption in the circadian rhythm was also observed in patients with major depressive disorder.^60^ Genes known to be crucial in the generation and regulation of circadian rhythm was found to be involved in depression.^61^ Clock genes dysregulation was assumed as an important factor associated with the development of both insomnia and depression.^62^ The probable regulatory mechanism is sleep disturbance and environmental factors cause the abnormal expression of clock genes, which in turn influence the mood symptoms and finally contribute to the happening of depressive episodes. A lot of studies were conducted to find significant correlation between SNPs (single nucleotide polymorphisms) of clock genes and depression; however, some of these experiments drew the exact opposite results, and most of them were failed.^63^, ^64^ One exception is SNP rs2287161 of Cryptochrome which might indicate a higher susceptibility to circadian dysregulation and MDD.^65^ The interruption of circadian rhythm may contribute to the development of sleep disturbance and depression, but many of the mechanisms by which the circadian system and sleep/wake cycles interact, or the precise molecular mechanisms between clock genes and depression remain elusive.

## 4 THE TREATMENT OF INSOMNIA DISORDER WITHOUT DEPRESSION

**Figure 2:**
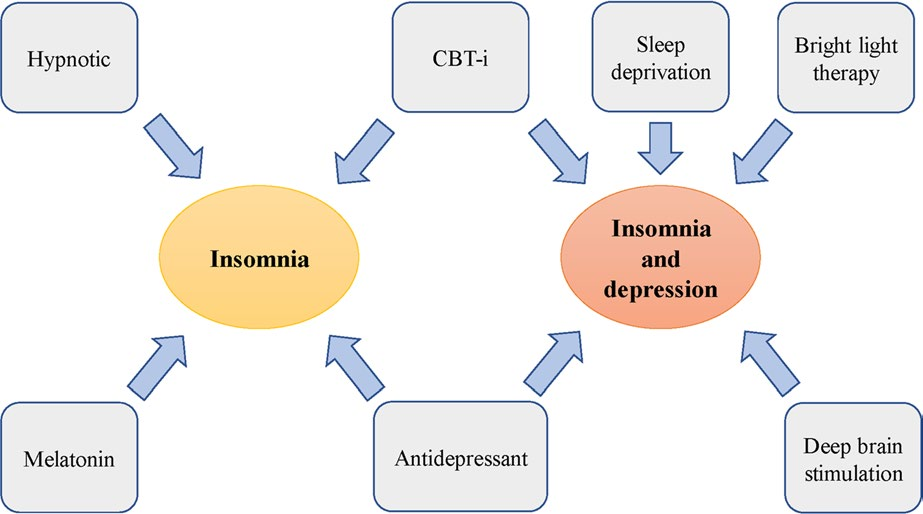
Summary of the treatments of insomnia with or without depression

As discussed before, sleep disturbances are an important risk factor for subsequent depression, and the treatment of sleep disturbance before depression is crucially meaningful. Cognitive‐behavioural therapy for insomnia (CBT‐i) has been recommended as the initial treatment for chronic insomnia disorder.^66^ CBT‐i therapy consists of a combination of treatments that include the following: (a) stimulus control, which aims to strictly limit the role of the bed (sleeping and sex) and restrict its association with stimulating behaviours; (b) sleep hygiene, which aims to develop a favourable sleep habit and create a comfortable environment that precedes sleep; (c) sleep restriction, which involves controlling the time spent in bed to improve sleep efficiency and thereby reinforce the ‘bed‐sleep connection’; (d) relaxation training, which is a series of practices that can help people to relax both mind and body before bedtime and (e) cognitive therapy, which offers education to change incorrect conceptions about sleep. In a high‐quality meta‐analysis, the authors explored the efficacy of CBT in patients with chronic insomnia symptoms. They concluded that CBT therapy was effective for sleep onset latency, wake after sleep onset and sleep efficiency at the post‐treatment time point and remained effective through late follow‐up.^67^ Another meta‐analysis focused on the effectiveness of CBT‐i in children and adolescents found that CBT‐i was instrumental in helping teenagers who were suffering from sleep complaints.^68^ In addition, sleep improvements following CBT‐i can last for 12‐36 months after treatment is finished.^13^, ^69^, ^70^ Furthermore, a blinded placebo‐controlled trial indicated that CBT was superior to zopiclone in the treatment of insomnia in old adults.^71^ In recent years, to generalize this idea, some alterations of traditional CBT‐i have been studied. Among them, group CBT‐i has been suggested as a mid‐level treatment in stepped care models, which showed significant improvements in sleep complaints and the effects remained persistent into follow‐up.^72^, ^73^ However, in clinical situations, pharmacotherapy for insomnia is widely used. In America, hypnotics were used to treat insomnia in approximately 6%–10% of adults in 2010.^74^ Approximately 90% of primary insomniac patients receive hypnotic prescriptions in Australia.^75^ The pharmacologic treatment of insomnia mainly includes hypnotic drugs (benzodiazepines [BZDs] and nonbenzodiazepines) and antidepressants. BZDs are a class of classic hypnotic drugs that includes lorazepam, triazolam, flurazepam, nitrazepam, diazepam and oxazepam. These drugs have been extensively used because of their satisfactory sedative and hypnotic effects. However, there has been a trend where these drugs are gradually being replaced by newer non‐benzodiazepines such as zaleplon, eszopiclone and zolpidem (the ‘Z‐drugs’), which may be due to the fatal drug poisoning of BZDs.^76^, ^77^ Zolpidem is a non‐benzodiazepine that is commonly used worldwide because of its good effect and low negative consequences compared with traditional BZDs.^78^, ^79^ However, a recent nationwide population‐based study found that there was a significant relationship between zolpidem use and suicide in people with or without comorbid mental disorders.^80^ This sounded an alarm for us, suggesting that non‐benzodiazepines may be not as good as we thought and that we should restrict the indications and avoid the abuse of this class of drug, even though the ‘Z‐drugs’ had been FDA approved hypnotics and recommended for the treatment of insomnia.^81^ Suvorexant is a new hypnotic that has been reported to be useful in the treatment of insomnia, but the relevant data are insufficient, and further well‐organized randomized controlled trials (RCT) are required to verify its usability and side effects (Figure 2).^82^, ^83^

Sedative‐hypnotic drugs are commonly used for the treatment of insomnia, but long‐term use of such drugs may lead to tolerance and even exacerbate sleep disturbances.^84^, ^85^ Sedating antidepressants at low dosages are often prescribed to insomnia patients. The TCA amitriptyline, trimipramine and doxepin, the serotonin antagonist and reuptake inhibitor trazodone and the tetracyclic antidepressant mirtazapine have been found to improve total sleep time and sleep efficiency and reduce wake after sleep onset and latency to persistent sleep.^76^, ^86^ However, due to the lack of randomized clinical trials, only doxepin is approved by FDA and recommended for the treatment of insomnia.^81^ Moreover, different antidepressants may in turn cause different sleep disturbances, such as RLS, sleep bruxism, REM behaviour disorder and nightmares, as well as weight gain, which is contraindicated in patients with obstructive sleep apnea.^66^, ^87^, ^88^, ^89^ In our opinion, sedative antidepressants are safe in low doses, for example, for doxepin as low as 3‐6 mg, which can be applied in patients when hypnotics are contraindicated, for example, elderly patients, patients with sleep apnea and patients with a history of alcohol and substance abuse.

Melatonin is a physiological hormone broadly used ‘off label’ to improve sleep complaints. A recent meta‐analysis showed that exogenous melatonin was useful in reducing sleep onset latency in primary insomnia.^90^ However, evidence for the therapeutic use of melatonin is still lacking. Therefore, the clinical practice guidelines do not suggest the use of melatonin as a treatment for insomnia.^81^ Further studies on the precise effect and mechanism of melatonin are warranted.

## 5 THE TREATMENT OF INSOMNIA DISORDER COMORBID WITH DEPRESSION

### 5.1 Pharmaceutical therapy

Patients with comorbid insomnia and depression tend to suffer more severe depressive symptoms, longer durations of treatment and lower rates of remission compared with depressed patients without sleep disturbance. Antidepressant drugs are generally prescribed to depressed patients, but some classes of antidepressants may exacerbate sleep quality, such as the SNRIs, monoamine oxidase inhibitors (MAOIs), NRIs, SSRIs and activating TCAs. According to studies with polysomnography, SSRIs, SNRIs and activating TCAs increase rapid eye movement sleep (REM sleep, REMS) latency, suppress REM sleep and impair sleep continuity. Sedating antidepressants decrease sleep latency, ameliorate sleep efficiency and increase slow wave sleep (SWS), with little effect on REMS.^91^ In this regard, sedating antidepressants is more favourable in patients with comorbid depression and sleep disturbance. Furthermore, sedating antidepressant treatment can significantly reduce the use of BZDs in patients with major depressed disorder,^92^ thereby greatly reducing the addiction to hypnotics. Antidepressant medications are typically the first‐line treatment for depression, but emerging drug resistance is of increasing concern. Agomelatine is a non‐sedative antidepressant drug with agonistic action at melatonergic MT1 and MT2 receptors and antagonistic action at serotonergic 5‐HT2c receptors, which can be a good choice for depressed patients with comorbid insomnia symptoms.^93^, ^94^ When compared to escitalopram, agomelatine can reduce sleep latency after both short‐term (after 2 weeks) and long‐term (after 24 weeks) treatment. And, in the next week, agomelatine slightly improved sleep continuity while escitalopram worsened sleep continuity.^95^ Furthermore, agomelatine increases the amount of SWS and improves daytime alertness without suppressing REM sleep.^96^ Thus, agomelatine may be a promising antidepressant drug that can solve both mood and sleep complaints.^94^, ^97^

### 5.2 CBT therapy

CBT is the most remarkable non‐pharmacologic treatment for insomnia disorders and is also useful for insomnia comorbid with depression. A recent meta‐analysis suggested that CBT‐i can improve sleep efficiency and achieve remission from insomnia when insomnia is comorbid with depressed disorders.^98^ In a 3‐year follow‐up study, it was indicated that treating insomnia was more helpful than treating depression; therefore, CBT therapy should also be performed in patients with cooccurring depression and insomnia.^99^ Another RCT targeting the effectiveness of CBT‐i in older adults with comorbid insomnia and depression demonstrated that CBT was effective at reducing both insomnia and depression severity in a 20‐week follow‐up.^100^ Although CBT is a promising therapeutic method for cooccurring insomnia and depression symptoms, there exist some limitations mainly because of a lack of professional psychiatrists, geographic distance to providers and the requirement of 6‐8 weeks of direct patient contact. These shortcomings can be solved by an advanced form of CBT‐i called internet‐delivered CBT or Digital CBT (DCBT). In this way, the idea of CBT can be disseminated more widely across a broader spectrum of the population. DCBT was proven to be effective in both insomnia and depression severity across diverse demographic groups, and the rates of remission following treatment were significantly high.^101^ Blom et al found that internet‐delivered CBT for insomnia was, in summary, more effective than internet‐delivered CBT for depression for adults comorbid with insomnia and depression.^102^ Moreover, patients receiving treatment for insomnia had a lower need for further insomnia treatment or medical prescriptions after treatment.^102^ In general, CBT treatment has had a definite effect on patients with depression and insomnia.

### 5.3 Combination therapy

For patients with depression and insomnia, people are more likely to focus the treatment on depressive symptoms and neglect the treatment of insomnia. However, several studies have shown that this approach is insufficient because residual insomnia is related to the relapse of depression. In clinical practice, adjunct measures are often used. Low‐dose trazodone or mirtazapine can be added to an SSRI, and MAOI for patients with coincident depression and sleep complaints.^103^ These sedative antidepressants can also be replaced by zaleplon, zolpidem or eszopiclone. Some studies have shown that eszopiclone coadministered with fluoxetine was associated with favourable sleep improvement and antidepressant response.^104^, ^105^ However, as clinical practitioners, we should pay close attention to the addiction and dependency of these sedative hypnotics because there is a relation between a history of high dosages of hypnotics and worse depression outcomes.^106^

Many studies have indicated that antidepressants plus CBT therapy were favourable in patients with comorbid insomnia and depression, although some of them did not reach a statistical difference. The combination of escitalopram and CBT showed better remission from both depression and sleep symptoms compared with escitalopram alone.^10^ A recent study compared the efficacy of CBT combined with antidepressants with standard antidepressant treatment in patients comorbid with insomnia and MDD reached the same conclusion.^107^ The favourable results in these studies are consistent with the perspective that the treatment of insomnia is as important as the treatment of depressive symptoms.

### 5.4 Sleep deprivation

Sleep deprivation (SD) is another kind of psychotherapy that typically involves depriving a patient of sleep for 36 consecutive hours (total sleep deprivation, TSD), and even a nap is not allowed. In this way, patients can reach a rapid improvement in an episode of depression, although the efficacy will disappear after the recovery sleep on the next day.^108^ To maintain the antidepressant effect, repeated sleep deprivation was implemented, but after restoring the usual sleep rhythm, the effect gradually diminished within a few weeks.^109^ When SD was combined with bright light therapy (BLT), a prolonged antidepressant effect was observed.^110^ Subsequent studies integrated pharmacology, TSD, sleep phase advance (SPA) and BLT, and they found that depressive symptoms were more reduced compared to pharmacological monotherapy and that the effect lasted longer, even in patients with drug‐resistant depression.^111^, ^112^, ^113^ Overall, due to the efficiency, simplicity and safety of SD, this can be a considerable treatment in clinical practice. Moreover, when combined TSD with SPA, pharmacology and BLT, it can be an effective way to treat drug‐resistant depression.

### 5.5 Deep brain stimulation

Deep brain stimulation is an emerging technology frequently used for movement disorders.^114^ Many studies have indicated that DBS is equally effective and safe in the treatment of treatment‐resistant depression (TRD).^115^, ^116^, ^117^, ^118^

However, a large heterogeneity in the clinical response to stimulation parameters was reported, and the samples in these studies were rather small. Despite this, DBS can be a new promising strategy for the treatment of patients with TRD.

## 6 CONCLUSION AND PROSPECTS

Sleep disturbance is not only a comorbidity of depression, but also a prodromal symptom, which can predict the occurrence and outcome of depression. It is important to highlight the treatment of sleep disturbance before, during and after depression. Hypnotics and antidepressants are generally applied for the treatment of patients comorbid with depression and sleep problems. However, hypnotics can cause depression, and the use of antidepressant can aggravate sleep disturbance. Alternative therapies such as CBT, SD and DBS were found to be effective in these patients. But the cure rate is low and the recurrence rate is rather high. A better understanding on the exact molecular mechanisms between sleep disturbances and depression is warranted, which can facilitate the management of coincident depression and sleep complaints quite a lot. More well‐organized RCTs are needed to verify the effectiveness of new drugs and non‐pharmacological therapies.

It is worth to mention that, in clinical context, sleep disturbance and depression are often comorbid with other mental health conditions, such as behaviour disorders, substance disorder and especially anxiety disorder.^119^ There is an intriguing interrelationship between anxiety and depression, and insomnia. Both depression and anxiety are related to future insomnia, and insomnia can lead to depression and anxiety in the future.^120^ Of note, the research on such relationships is crucial not only because it may help to prevent future conditions but also because comorbid problems are usually much more difficult to treat and indicate poorer prognosis.

## CONFLICT OF INTEREST

The authors declare that the research was conducted in the absence of any commercial or financial relationships that could be construed as a potential conflict of interest.
